# Determination of Temperature-Dependent Coefficients of Viscosity and Surface Tension of Tamarind Seeds (*Tamarindus indica* L.) Polymer

**DOI:** 10.3390/polym13040610

**Published:** 2021-02-18

**Authors:** Rishabha Malviya, Sheetal Jha, Neeraj Kumar Fuloria, Vetriselvan Subramaniyan, Srikumar Chakravarthi, Kathiresan Sathasivam, Usha Kumari, Dhanalekshmi Unnikrishnan Meenakshi, Omji Porwal, Akanksha Sharma, Darnal Hari Kumar, Shivkanya Fuloria

**Affiliations:** 1 Department of Pharmacy, SMAS, Galgotias University, Greater Noida, Gautam Buddh Nagar 201310, India; rishabha.malviya@galgotiasuniversity.edu.in (R.M.); sheetu.jha92@gmail.com (S.J.); akanksha_sharma.smasmop@galgotiasuniversity.edu.in (A.S.); 2 Faculty of Pharmacy, AIMST University, Kedah 08100, Malaysia; 3 Faculty of Medicine, Bioscience and Nursing, MAHSA University, Kuala Lumpur 42610, Malaysia; drvetriselvan@mahsa.edu.my (V.S.); srikumar@mahsa.edu.my (S.C.); 4 Faculty of Applied Science, AIMST University, Kedah 08100, Malaysia; skathir@aimst.edu.my; 5 Faculty of Medicine, AIMST University, Kedah 08100, Malaysia; usha_harischandran@aimst.edu.my; 6 College of Pharmacy, University of Science and Technology, Muscat 130, Oman; dhanalekshmi@omc.edu.om; 7 Department of Pharmacognosy, Tishk International University, Erbil 44001, Iraq; omji.porwal@tiu.edu.iq; 8 Jeffrey Cheah School of Medicine and Health Sciences, Monash University Malaysia, Selangor 47500, Malaysia; hari.kumar@monash.edu

**Keywords:** tamarind seed polymer, viscosity, temperature, surface tension, polysaccharide

## Abstract

The rheological properties of tamarind seed polymer are characterized for its possible commercialization in the food and pharmaceutical industry. Seed polymer was extracted using water as a solvent and ethyl alcohol as a precipitating agent. The temperature’s effect on the rheological behavior of the polymeric solution was studied. In addition to this, the temperature coefficient, viscosity, surface tension, activation energy, Gibbs free energy, Reynolds number, and entropy of fusion were calculated by using the Arrhenius, Gibbs–Helmholtz, Frenkel–Eyring, and Eotvos equations, respectively. The activation energy of the gum was found to be 20.46 ± 1.06 kJ/mol. Changes in entropy and enthalpy were found to be 23.66 ± 0.97 and −0.10 ± 0.01 kJ/mol, respectively. The calculated amount of entropy of fusion was found to be 0.88 kJ/mol. A considerable decrease in apparent viscosity and surface tension was produced when the temperature was raised. The present study concludes that the tamarind seed polymer solution is less sensitive to temperature change in comparison to *Albzia lebbac* gum, *Ficus glumosa* gum and *A. marcocarpa* gum. This study also concludes that the attainment of the transition state of viscous flow for tamarind seed gum is accompanied by bond breaking. The excellent physicochemical properties of tamarind seed polymers make them promising excipients for future drug formulation and make their application in the food and cosmetics industry possible.

## 1. Introduction

In recent decades, natural polymers, compared to synthetic polymers, have offered more beneficial effects and are used in the food industry, the pharmaceutical industry, cosmetics, and other sectors [1,2]. The polymers derived from plants demonstrate a wide range of applications in the design of formulation and dispersion systems and are mainly found in thickening systems. Important developments in tissue engineering and diagnostic and therapeutic techniques have been made via the use of polymers in various fields of modern medicine. Polymers have been used to monitor drug release rates, avoid toxicity, protect drugs from pre-delivery degradation, target drugs at the site of action, and enhance absorption, bioavailability, and therapeutic effectiveness [3]. Chitosan is desirable in cancer therapy applications, as it has shown anti-tumor activity [4]. The various natural polymers possessing this property include xanthan gum, locust bean gum, and guar gum. Another plant-derived polymer possessing this property is tamarind seed polymer [5]. *Tamarindus indica* L. (Tamarind), commonly called Imli in India and Asam Jawa in Malaysia, has high potential in the pharmaceutical industry. Tamarind seed yields tamarind seed gum, a rich source of polysaccharides containing xylose, glucose, and galactose in a ratio of 3:2:1 [6,7,8]. The tamarind seed polymer (TSP) isolation and characterization involve a simple and cost-effective method. The TSP is a polysaccharide of high molecular weight that, when dissolved in water, forms a viscous consistency. Based on its multi-functional potential, TSP can be concluded as a promising natural polymer that offers a wide variety of applications [9]. This Leguminosae family compound is widely indigenous to India, Myanmar, Bangladesh, Malaysia, Thailand, and Sri Lanka [10]. TSP is obtained from the seeds of kernels. In Asian countries, it is mainly grown and used as a gel and binder in tablet dosage form [11].

For the commercialization of TSP in the food and pharmaceutical industry, it is important to assess its rheological properties. The rheological study is based on the material that exhibits elastic, viscous, and plastic behavior under different conditions of flow. The components are generally known as soft matter or complex fluids that include polymers, suspensions, emulsions, gels, and inks. [12]. The rheological property measurement is suitable for a range of materials, starting from fluids such as surfactants, and polymers dilute solutions to different formulations such as semisolid preparations, gels, creams molten, or solid polymers. It is used to understand the fundamental essence of a system (basic science), quality control (raw materials and products, process), and the effect of various parameters on product quality. The study suggests that an assessment of the rheological property is based on the determination of the viscosity and surface tension of the solution. In the year 1844, Hagen and Poiseuille worked on the flow of liquid from tubes and proposed an equation for the viscosity of liquids. The equation is known as Poiseuille’s equation. Similarly, the surface tension was determined by calculating the Eotvos equation [13]. 

To determine rheological characteristics, polymers should be prepared in an aqueous solution, and certain instruments are needed to do so. The viscosity and surface tension of the aqueous solution of polymers can be determined using the Ostwald viscometer and stalagmometer [14]. Both the surface tension and viscosity are determined at different temperature intervals. Based on the scientific evidence, the present study was designed to determine the temperature-dependent coefficients of viscosity and the surface tension of tamarind seed polymer.

In the form of macromolecules, natural polymers are obtained. The increasing demand for natural polymers in households, the food industry, agriculture, and packaging also helps to minimize environmental pollutions and eradicate landfills. Natural polymers are non-toxic, biodegradable, and biocompatible and swell as they come into contact with aqueous media, so they have been used in the preparation of dosage forms for controlled release or sustained release [15]. Gums are important additives used in the food industry, the dairy industry, and bakery products. They are used as thickeners and emulsifiers in the food and dairy industry and as adhesives and viscosity enhancers in bakery products. In the pharmaceutical industry, they are used as binders and coating material in tablet dosage forms and as suspending and emulsifying agents in liquid dosage forms. In the cosmetic industry, gums are used as adhesives and provide a smooth texture. Plant gums are also used as protective colloids and flocculants in the paint industry, the finishing agent in the printing industry, carbonless paper in the paper industry, protective in the ink industry, and adhesive in the glass and metal industry [9,16]. A search for alternative sources of natural gum is required to reduce the dependency upon pre-established natural gums for commercial applications. Characterization in terms of viscosity is the most important parameter of gum for commercial applications. An adhesive nature and viscosity are intrinsic properties of gums, which makes them suitable candidates as viscosity enhancers and binders for various industrial applications. To search and characterize a natural gum for industrial applications, investigators have selected the tamarind seed gum, because it is available as waste after utilizing the pulp of some fruit (as a flavoring agent in the food industry) and has been further characterized in terms of viscosity [17]. During the preparation of cosmetics and bakery and dairy products, a high temperature is required to mix all ingredients properly, as the mixed ingredients need to maintain an appropriate viscosity during preparation. Therefore, an investigation of temperature-dependent viscosity is necessary. This investigation is applied to the TSP to determine its efficacy during the formulation of different products.

The TSP is used as a thickening, gelling, and stabilizing agent in food. In pharmaceutical tablets, these polymers can also serve as a binder, humectant, and emulsifier in various forms of the formulation. This reveals that the tamarind polymer is highly viscous, mucoadhesive, and biocompatible [9]. The tamarind gum has the largest scope in the pharmaceutical industry and serves as a binder in tablet formulation, ocular drug delivery systems, and drug delivery systems for prolonged release [16].

The tamarind seed polymer is used as a binder for tablet dosage forms. The TSP can also be used as a potential candidate for lower gastrointestinal tract targeted drug delivery [18].

## 2. Materials and Methods 

### 2.1. Purification of Tamarind Seed Polymer

*Tamarindus indica* seeds were bought from a local market in Greater Noida, India. Distilled water was used as a solvent for the preparation of an aqueous solution of the TSP in the laboratory. The desired amount of purified tamarind seeds was dried at 40 °C for 10 min, and brown coatings were removed and powdered. Further, distilled water was added to the beaker containing the seed powder and stirred for a few hours at 40 °C to prepare the slurry. The slurry was then kept under a mechanical stirrer to form a uniform size distribution. Again, by adding the required amount of distilled water using a graduated cylinder, the beaker was kept on the magnetic stirrer for 1 h at 40 °C until the gum was dissolved homogeneously. It was then filtered using a muslin cloth, and the precipitate was dried in an oven at 40 °C. The size of the dried product was reduced by using a domestic mixer and grinder, and powder was passed from a #45 mesh. The powder then underwent various tests and was kept in an airtight container.

### 2.2. Temperature-Dependent Characterization of Tamarind Seed Polymer

Before viscosity determination, the TSP was dissolved in an aqueous solution and filtered. The solution of TSP was prepared by dissolving the polymer into distilled water at 40 °C for 60 min at 50 rpm. To evaluate the effect of temperature, a 1% *w*/*v* solution of the TSP was prepared. The pH of the solution was also determined, and the result was shown as the average of the triplicate study with a standard deviation. To carry out the viscosity measurements, an Ostwald viscometer was used. A relative density bottle was used to determine the density of the polymer solution. To determine the thermodynamic parameter, a calibrated laboratory-scale thermometer was used. The time was noted in triplicate, and the viscosity was then calculated.

To determine the viscosity of the TSP solution, different thermodynamic parameters were evaluated. The effect of temperature on viscosity was correlated using the Arrhenius Equation (1) [19].

η = A exp [EF/RT](1)

where T is temperature, A is the Arrhenius coefficient, η is the apparent viscosity, and R is the universal gas constant. Equation (1) can be further expressed in logarithmic form to determine different parameters. The thermodynamic parameter of the viscosity was also determined using Frenkel–Eyring Equation (2). The equation was developed by Meredith Gwynne Evans, Henry Eyring, and Michael Polanyi in 1935. The equation derives from the theory of the transition state and is equivalent to the empirical, thermodynamics-derived Arrhenius equation. Frenkel–Eyring Equation (2) is given as follows:

ln (η/T) = (ln A − ∆SV/R) + ∆HV/RT(2)

where R is the universal gas constant, A is the pre-exponential factor, T is the absolute temperature, ∆SV is the change in entropy, and ∆HV is enthalpy changes of viscous flow. 

The Gibbs–Helmholtz equation is a thermodynamic equation that was used as a function of temperature to determine changes in the Gibbs energy of a system. It is named after Hermann von Helmholtz and Josiah Willard Gibbs. Gibbs–Helmholtz Equation (3) is given as follows:

∆Gv = ∆Hv − T∆Sv(3)

where ∆G is Gibb’s free energy, ∆ Hv is the enthalpy change, and ∆ Sv is entropy change. Similarly, Eotvos Equation (4) was calculated to determine the surface tension. Equation (4) is given as follows:

γ V^2/3^ = K (Tc − T) (4)

where V is the liquid molar volume, Tc is the critical temperature, and K is the Eotvos constant valid for approximately all substances. K = 2.1 × 10^−7^ [J K^−1^ mol^−2/3^]. For water, V = 18 mL/mol, and Tc = 647 K (347 °C). The temperature coefficient is unusually smaller for water, alcohol, and other associated liquids but is always negative for a pure substance.

## 3. Results and Discussion

As discussed by the author in previous studies, the major component of tamarind seed pulp is carbohydrate (70.8%), followed by protein (3.1%) and fiber (3.0%). The TSP consists of an ‒OH group attached with a cyclic structure. Galactose, xylose, and glucose are present in a 3:1:2 molar ratio. The ratio predicts that galactose is a major component of the TSP followed by glucose and xylose [20]. As described by authors elsewhere, the O-H stretch (3508.70 cm^−1^), C-H stretch (2925.81 cm^−1^), C-H rock (1384.79 cm^−1^), and C-H bend (1332.72 cm^−1^) peaks were observed in the IR spectra of the TSP [21]. The results of the IR spectral study were also supported by the study performed by Chawananorasest et al., showing an average molecular weight of the TSP ranging from 700 to 880 kDa [22]. 

### 3.1. Effect of Temperature on Viscosity

Based on the experimental protocol followed in Section 2, the temperature effect on the viscosity of the TSP was observed (pH: 6.5 ± 0.01). Generally, the viscosity of fluid decreases with an increase in temperature. The liquid viscosity depends upon the strength of the attractive forces between the molecules and directly depends on the molecules’ composition, size, shape, and kinetic energy, which further depends on the temperature [23]. This is based on the fact that, when the temperature of the system is raised, energy is effectively added, which gives the molecules of the liquid the required energy to overcome the intermolecular force. Due to this, the molecules further move apart, and this leads to a decrease in the viscosity of the liquid [24]. This happened with the tamarind seed polymer (TSP) solution. When the temperature was raised, the viscosity of the TSP gradually decreased.

**Figure 1:**
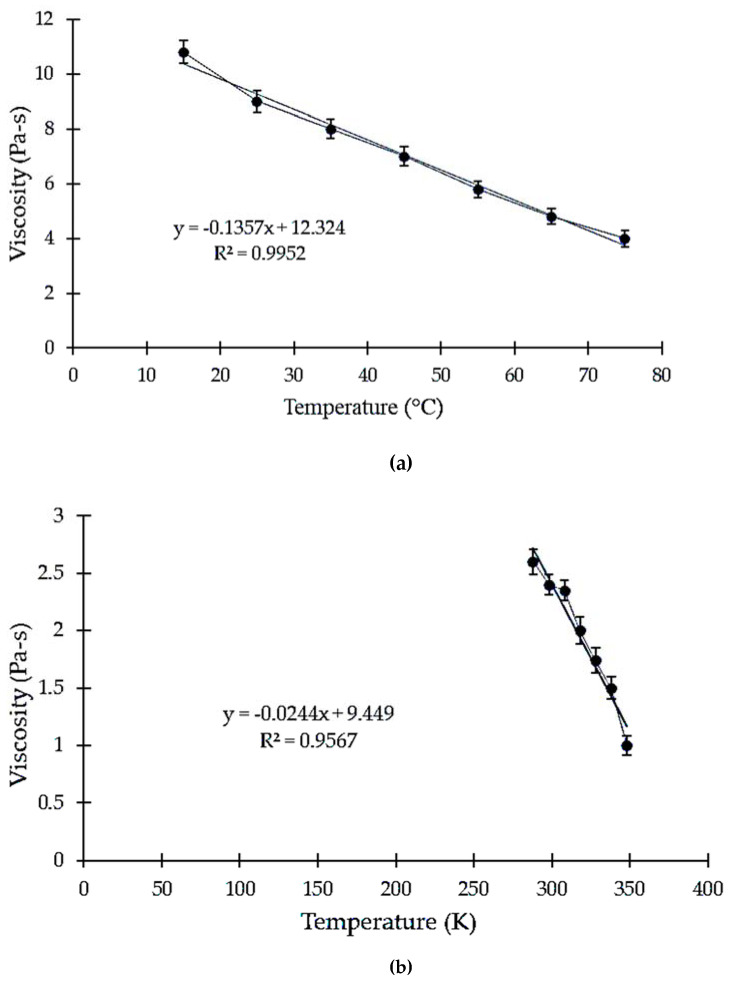

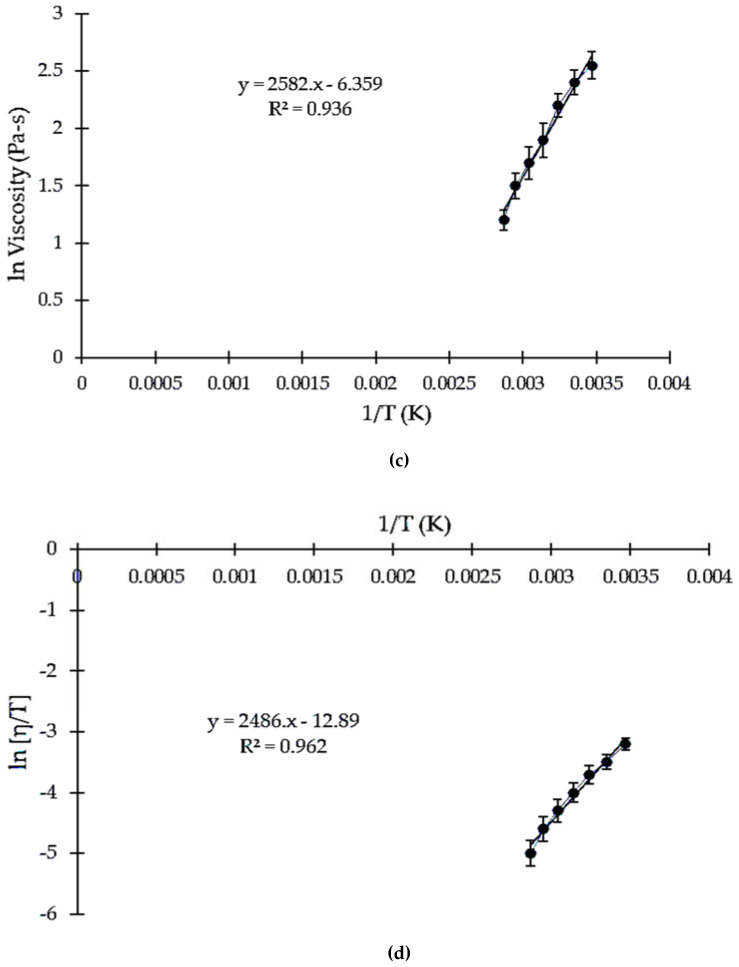
(**a**) The variation in viscosity with temperature (°C), (**b**) the effect of temperature (K) on the ln viscosity of the solution, (**c**) the variation in ln viscosity with temperature (K), and (**d**) the variation in ln [η/T] with temperature (1/T).

A graph was plotted showing the relationship between the logarithmic viscosity and the reciprocal of the temperature (K). The slope so obtained helps to determine the different temperature-dependent parameters, including the Arrhenius parameters. In Figure 1a–d, it is obvious that the viscosity of tamarind seed gum reduces with an increase in temperature. Using the graph slope, the activation energy can be easily calculated. From the viscosity results, the activation energy of the viscous flow (EF) of the gum was determined using the Arrhenius equation [25]. From both sides of the logarithm of Equation (1), a new equation was derived that yielded Equation (2) in the form of y = mx + c. This form of the Arrhenius equation can be used to determine the slope and y-intercept from an Arrhenius plot. It makes it very easy to note that, as temperature increases, the rate constant decreases, according to the graph. Therefore, it can be inferred that the rate constant is inversely proportional to the temperature. The results obtained indicate a linear relationship between ln [n] and 1/T.

The slope of the plot is equal to EF/2.303 R, from which EF was evaluated. The plot reveals a high degree of linearity, i.e., R^2^ = 0.936. The value of the EF of the gum was found to be 20.46 ± 1.06 kJ/mol. This value is within the range of values recorded by de Paula and Rodrigues for certain plant gums, including a gum with an EF value of 16.2 kJ/mol [26], *A. marcocarpa* gum with an EF value of 16.8 kJ/mol calculated by Silva et al. [27], and *Albzia lebbac* gum with an EF value of 15.9‒17.2 kJ/mol [28]. In general, higher EF values indicate that the polymer solution is less vulnerable to temperature changes and vice versa [29,30]. A low EF value denotes the existence of few intra- and intermolecular interactions in the polymer or gums being studied. In a study, the investigator showed that *Ficus glumosa* gum showed a very low EF value (1.915 kJ/mol) as compared to the EF value of Arabic gum (15 kJ/mol) [31]. The EF value of the gum in the present study was higher than the EF value of the Arabic gum and *Ficus glumosa* gum [31]. Therefore, based on the EF value, the gum used in the present study is deemed to be more suitable than *Albzia lebbac* gum, *Ficus glumosa* gum, and *A. marcocarpa* gum for commercial applications. 

**Figure 2:**
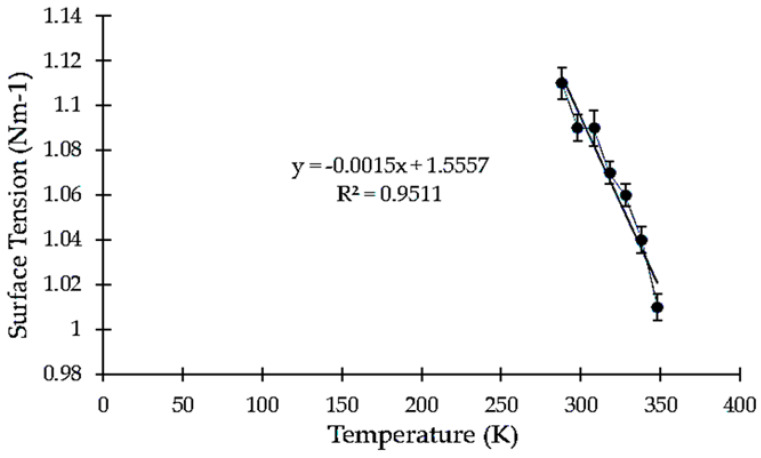
Variation in surface tension with respect to temperature.

The EF values for polymers were strongly bonded by inter- and intramolecular interactions and were found to be 27 kJ/mol or higher [32]. The Frenkel–Eyring equation was used to calculate the thermodynamic parameter of the viscosity [33]. Frenkel–Eyring Equation (5) is given as follows:

ln (η/T) = (ln A − ∆SV/R) + ∆HV/RT(5)

where A is the pre-exponential factor, T is absolute temperature, ∆SV is the change in entropy, R is the universal gas constant, and ∆HV is the enthalpy change in viscous flow. In Equation (3), a plot of ln (η/T) versus 1/T is estimated to be linear (as shown in Figure 2) with slope and intercept equal to ∆HV/RT and (ln A − ∆SV/R). Figure 2 shows a high degree of linearity, where the value of R^2^ = 0.962. The values of ∆HV and ∆SV were calculated to be 23.66 ± 0.97 and −0.10 ± 0.01 kJ/mol, respectively. 

In one investigation, it was shown that the gum extracted from wild sage seed had a ∆GV of 0.14–2.44 kJ/mol, a ∆SV value of 6.3–52.2 J/mol, and a ∆HV value of 0.52–14.99 kJ/mol [34].

In another study, the investigator extracted the mucilage from *Chrysophyllum lanceolatum* (blume) dc fruits, and the ∆HV was found to be 0.258 kJ/mol [35], which is slightly lower than the ∆HV calculated in the present investigation. Salehi et al. extracted polysaccharides from basil seeds and a polysaccharide solution, and the ∆SV, ∆HV, and ∆GV were 8.12–33.2 J/mol, 0.62–7.8 kJ/mol, and 1.62–4.42 kJ/mol, respectively [36]. The value of ∆GV determined for basil seed is lower than that obtained in the present study for the TSP (55.46 ± 1.69 kJ/mole).

Since the changes in entropy and enthalpy values are negative and positive, respectively, it can be determined that bond breaking follows the attainment of the transition state of viscous flow for tamarind seed gum. The negative value of the change in entropy is often correlated with the uncoiling and orientation of the polymer molecules, and the system becomes more orderly in the course of flow [37]. The free energy of activation of flow is calculated by substituting the value of change in enthalpy and entropy into the Gibbs–Helmholtz equation. The Gibbs–Helmholtz equation, named after Hermann von Helmholtz and Josiah Willard Gibbs, is a thermodynamic equation used as a function of temperature to calculate changes in the systems of Gibbs energy. 

With Equation (3), the ∆GV was calculated. The calculated value of ∆GV (55.46 ± 1.69 kJ/mole) is relatively low and confirms the existence of few intra- and intermolecular interactions. In fluid mechanics, the Reynolds number is the ratio of inertial forces between viscous forces and thus quantifies the relative importance of the flow conditions of these types of forces. It helps to assume similar flow patterns in various fluid flow situations. George Gabriel Strokes proposed the Reynolds number, which can be represented as Equation (6) [38]:

η = R exp [αT](6)

where α is a constant, R is the Reynolds number, and η is the apparent viscosity. From the graph between ln viscosity and temperature, the Reynolds number and α were determined. 

### 3.2. Effect of Temperature on Surface Tension

The surface tension of the TSP decreases with an increase in temperature. With the temperature rise, the molecules’ kinetic energy also increases. Therefore, the strength of intermolecular forces decreases, which also results in the decrease in surface tension. The effect of temperature over the surface tension is displayed in Figure 2.

Eotvos Equation (4) was used to determine the surface tension. The value of K was found to be 2.1 × 10^−7^ [J K^−1^ mol^−2/3^]. For water, V and Tc were estimated as 18 mL/mol and 647 K (347 °C), respectively. The surface tension was found to be 3.38 ± 0.33 N m^−1^ × 10^−7^. In general, the temperature coefficient for alcohols, water, and other related liquids is slightly lower, but still negative for pure substances [39]. Fusion entropy increases the entropy of a substance while melting. As the degree of disorder rises in the transition from an ordered solid to a disorganized liquid structure, this is always positive. Fusion is a first-order phase change that occurs at a constant temperature [40]. Equations (7) and (8) were used to calculate the entropy of fusion (∆S). The entropy of fusion (∆S) is expressed in J mol^−1^ K^−1^. It is expressed as Equation (9).

∆G_fus_ = ∆H_fus_ − T × ∆S_fus_ < 0 (7)

Here, ∆G is the Gibbs free energy, ∆H_fus_ is the fusion enthalpy, T is the temperature, and ∆S_fus_ is the entropy of fusion. At equilibrium, temperature is equal to the melting point, i.e., T = Tf [41].

∆G_fus_ = ∆H_fus_ − T × ∆S_fus_ = 0 (8)

From Equation (9), it is concluded that the entropy of fusion is the heat of fusion divided by the temperature in Kelvin:

∆S_fus_ = ∆H_fus_/Tf (9)

where S is the entropy of fusion, H is the enthalpy of fusion, and T is fusion temperature. The entropy of fusion was found to be 0.86 ± 0.03 kJ mol^−1^ K^−1^. Table 1 summarizes the evaluated parameters of the gums and their results.

Paula et al. studied the composition and rheological properties of *Albizia lebbeck* gum and determined its EF values, which were found to be 16.6 and 17.2 kJ mol^−1^ [42], similar to the present study’s EF value, which was found to be 20.46 ± 1.06 kJ/mol. Varma et al. determined that *Albizia lebbeck* gum was used in the formulation of pH-sensitive drug-releasing O/W emulsions, which shows that the TSP can also be used in the preparation of pharmaceutical formulations [43]. Wientjes et al. have determined the activation energy of guar gum, which was found to be 10.0 kJ mol^−1^ [44]. A commercial guar gum product was prepared—60 capsules that are sold under the brand Swanson and used for the treatment of digestive system problems. Both studies proved that the TSP, which has an activation energy of 20.46 ± 1.06 kJ/mol, which is similar to the above studies, can also be used for commercial product preparation. 

## 4. Conclusions

The rheological properties of aqueous solutions of tamarind seed gum were determined at different temperatures, and it was noted that the viscosity and surface tension of tamarind seed polymer decrease with an increase in temperature. The temperature coefficient of viscosity and surface tension of tamarind seed polymer is calculated. From this study, it was concluded that the tamarind seed polymer solution is less sensitive to change in temperature. It can also be concluded that the attainment of the transition state of viscous flow for tamarind seed gum is accompanied by bond breaking. A negative value of entropy change is correlated with the uncoiling and orientation of the polymer molecules, and the system becomes more ordered in the course of flow. Tamarind seed polymer possesses a wide range of properties, which makes it a promising excipient for future formulations of drugs and for finding possible applications in the food and cosmetics industries as well. The present research concludes that the tamarind seed polymer solution is less vulnerable to temperature variations. This research also establishes that bond breaking follows the achievement of the transition state of viscous flow for tamarind seed gum. The physicochemical properties of TSP demonstrate it as a successful excipient that can be used in the food and cosmetics industry and in drug formulation. 

## Figures and Tables

**Table 1:** Evaluated parameters of gum.

S.No.	Parameters	Results
1.	Activation energy	20.46 ± 1.06 kJ/mol
2. 3. 4. 5.	Change in entropy Change in enthalpy Entropy of fusion Gibb’s free energy	23.66 ± 0.97 kJ/mol 0.10 ± 0.01 kJ/mol 0.86 ± 0.03 kJ mol^−1^ K^−1^ 55.46 ± 1.69 kJ/mole

## Data Availability

The data presented in this study are available on request from the corresponding author.

## References

[1] Alonso-Sande M., Teijeiro D., Remunan-Lopez C., Alonso M.J. (2009). Glucomannan a promising polysaccharide for biopharmaceutical purposes. Eur. J. Pharm. Biopharm..

[2] Satturwar P.M., Fulzele S.V., Dorle A.K. (2003). Biodegradation and *in vivo* biocompatibility of rosin: A natural film-forming polymer. AAPS Pharm. Sci. Tech..

[3] Carmen Chifiriuc M., Mihai Grumezescu A., Grumezescu V., Bezirtzoglou E., Lazar V., Bolocan A. (2014). Biomedical applications of natural polymers for drug delivery. Curr. Org. Chem..

[4] De Souza R., Zahedi P., Allen C.J., Piquette-Miller M. (2010). Polymeric drug delivery systems for localized cancer chemotherapy. Drug Deliv..

[5] Saettone M.F., Burgalassi S., Giannaccini B., Boldrini E., Bianchini P., Luciani G. (1997). Ophthalmic Solutions Viscosified with Tamarind Seed Polysaccharide. U.S. Patent.

[6] El-Siddig K.E., Gunasena H.P.M., Prasad B.A., Pushpakumara D.K., Ramana K.V.R., Vijayanand P. (2006). Tamarind: Tamarindus Indica.

[7] Freitas R.A., Martin S., Santos G.L., Valenga F., Buckeridge M.S., Reicher F. (2005). Physico-chemical properties of seed xyloglucans from different sources. Carbohydr. Polym..

[8] Singh R., Malviya R., Sharma P.K. (2011). Extraction and characterization of tamarind seed polysaccharide as a pharmaceutical excipient. Pharmacogn. J..

[9] Malviya R., Srivastava P., Bansal M., Sharma P.K. (2010). Formulation, evaluation and comparison of sustained release matrix tablets of diclofenac sodium using tamarind gum as release modifier. Asian J. Pharm. Clin. Res..

[10] Glicksman M., Glicksman M. (1996). Tamarind seed gum. Food Hydrocoll.

[11] Srivastava P., Malviya R., Gupta S., Sharma P.K. (2010). Evaluation of various natural gums as release modifiers in tablet formulations. Phcog. J..

[12] Larson R.G. (1999). The Structure and Rheology of Complex Fluids.

[13] Rouillard E.E.A. (1985). A Study of Boiling Parameters under Conditions of Laminar Non-Newtonian Flow with Particular Reference to Massecuite Boiling. Ph.D. Thesis.

[14] Hapanowicz J. (2020). Proposition of non-standard method useful for viscosity measurements of unstable two-phase systems coupled with examples of its application. Measurement.

[15] Langer R.S., Peppas N.A. (1981). Present and future applications of biomaterials in controlled drug delivery systems. Biomaterials..

[16] Ghelardi E., Tavanti A., Celandroni F., Lupetti A., Blandizzi C., Boldrini E., Campa M., Senesi S. (2000). Effect of a novel mucoadhesive polysaccharide obtained from tamarind seeds on the intraocular penetration of gentamicin and ofloxacin in rabbits. J. Antimicrob. Chemother..

[17] Stokes G. (1851). On the effect of the internal friction of fluids on the motion of pendulums. Trans. Camb. Philos. Soc..

[18] Shukla A.K., Bishnoi R.S., Kumar M., Fenin V., Jain C.P. (2018). Applications of tamarind seeds polysaccharide-based copolymers in controlled drug delivery: An overview. Asian J. Pharm. Pharmacol..

[19] Malviya R., Sharma P.K., Dubey S.K. (2019). Characterization of neem (*Azadirachita indica*) gum exudates using analytical tools and pharmaceutical approaches. Curr. Nutr. Food Sci..

[20] Katiyar N., Malviya R., Sharma P.K. (2014). Pharmaceutical applications and formulation based patents of *Tamarindus indica* seed polysaccharide and their modified derivatives. Adv. Biol. Res..

[21] Verma S., Bansal J., Kumar N., Malviya R., Sharma P.K. (2014). Isolation and characterization studies of mucilage obtained from *Trigonella foenum greacum l.* seed and *Tamarindus indica* polysaccharide as a pharmaceutical excipient. J. Drug Deliv. Ther..

[22] Chawananorasest K., Saengtongdee P., Kaemchantuek P. (2016). Extraction and characterization of tamarind (*Tamarind indica* L.) seed polysaccharides (TSP) from three difference sources. Molecules.

[23] Anema S.G., Lowe E.K., Li Y. (2004). Effect of pH on the viscosity of heated reconstituted skim milk. Int. Dairy J..

[24] Lee D.W., Ruths M., Israelachvili J.N. (2017). Surface forces and nanorheology of molecularly thin films. Nanotribology and Nanomechanics.

[25] Malviya R., Sharma P.K., Dubey S.K. (2017). Kheri (*Acacia chundra*, family: Mimosaceae) gum: Characterization using analytical, mathematical and pharmaceutical approaches. Polim. Med..

[26] Paula R.C.M., Rodrigues J.F. (1995). Composition and rheological properties of cashew tree gum, the exudates polysaccharide from *Anacardium occidentale*. Carbohydr. Polym..

[27] Silva A.G., Rodrigues J.F., De Paula R.C.M. (1998). Structure-property relationships in food biopolymer gels and solutions. Polimeros.

[28] Menon A.R.R. (2003). Melt rheology of ethylene propylene diene rubber modified with propylene phosphorylated cashew nut shell liquid prepolymer. Iran. Polym. J..

[29] Shaikh M., Shafique M., Aggarwal B.R., Aeooqui M.F. (2011). Density, viscosity and activation parameters of viscous flow for cetrimide in ethanol+water system at 301.5 K. Rasayan. J. Chem..

[30] Nair S.V., Oommen Z., Thomas S. (2002). Melt elasticity and flow activation energy of nylon 6/polystyrene blends. Mater. Lett..

[31] Ameh P.O. (2013). Physicochemical properties and rheological behaviour of *Ficus glumosa* gum in aqueous solution. Afr. J. Pure Appl. Chem..

[32] Eddy N.O., Udofia I., Uzairu A., Ongenyi A.O., Obadimu C. (2014). Physiochemical, spectroscopic and rheological studies on *Eucalyptus citriodora (ec)* gum. J. Polym. Biopolym. Phys. Chem..

[33] Acevedo I.L., Kartz M. (1990). Viscosities and thermodynamics of various flows of some binary mixtures at different temperatures. Solut. Chem..

[34] Salehi F., Kashaninejad M. (2014). Kinetics and thermodynamics of gum extraction from wild sage seed. Int. J. Food Eng..

[35] Boruah A.K., Nath L.K. (2016). Extraction, purification and physicochemical evaluation of mucilage of *Chrysophyllum lanceolatum* (blume) dc fruits: An investigation for bioadhesive property. Int. J. Pharm. Pharm. Sci..

[36] Salehi F., Kashaninejad M., Tadayyon A., Arabameri F. (2015). Modeling of extraction process of crude polysaccharides from basil seeds (*Ocimum basilicum* L.) as affected by process variables. J. Food Sci. Technol..

[37] Eddy N.O., Ameh P.O., Gimba C.E., Ebenso E.E. (2013). Rheological modeling and characterization of *Ficus platyphylla* gum exudates. J. Chem..

[38] Rott N. (1990). Note on the history of the reynolds number. Annu. Rev. Fluid Mech..

[39] Tu W., Chen Z., Gao Y., Li Z., Zhang Y., Liu R., Tian Y., Wang L.M. (2014). Glass transition and mixing thermodynamics of a binary eutectic system. Phys. Chem. Chem. Phys..

[40] Manjunath M., Gowda D.V., Kumar P., Srivastava A., Osmani R.A., Shinde C.G. (2016). Guar gum and its pharmaceutical and biomedical applications. Adv. Sci. Eng. Med..

[41] Papon P., Leblond J., Meijer P.H. (2002). Physics of Phase Transitions.

[42] De Paula R.C.M., Santana S.A., Rodrigues J.F. (2001). Composition and rheological properties of *Albizia lebbeck* gum exudate. Carbohydr. Polym..

[43] Varma C.A.K., Kumar K.J. (2018). Formulation and optimization of pH sensitive drug releasing O/W emulsions using *Albizia lebbeck* L. seed polysaccharide. Int. J. Biol. Macromol..

[44] Wientjes R.H., Duits M.H., Jongschaap R.J., Mellema J. (2000). Linear rheology of guar gum solutions. Macromolecules.

